# Characteristics of non-neoplastic epithelium that appears within gastric cancer with and without *Helicobacter pylori* eradication: A retrospective study

**DOI:** 10.1371/journal.pone.0248333

**Published:** 2021-03-10

**Authors:** Hiroto Noda, Mitsuru Kaise, Ryuichi Wada, Eriko Koizumi, Kumiko Kirita, Kazutoshi Higuchi, Jun Omori, Teppei Akimoto, Osamu Goto, Hiroshi Kawachi, Katsuhiko Iwakiri

**Affiliations:** 1 Departments of Gastroenterology, Nippon Medical School Hospital, Tokyo, Japan; 2 Departments of Pathology, Nippon Medical School Hospital, Tokyo, Japan; 3 Department of Pathology, The Cancer Institute Hospital of Japanese Foundation for Cancer Research (JFCR), Tokyo, Japan; University of Malaya Faculty of Medicine, MALAYSIA

## Abstract

A non-neoplastic epithelium (NE) often appears in gastric cancer (GC). We explored the histological features of NE in comparison between HP-eradicated and HP-infected GCs. We enrolled 40 HP-eradicated and 40 HP-infected GCs matched by size, macroscopic and histological type. NE was classified into full gland type and surface type; the former was a non-neoplastic gland isolated within cancer, and the latter was NE on the surface of the cancer. Surface type was additionally divided into NE at the cancer margin (marginal surface type) and NE inside cancer (internal surface type). The primary endpoints were the frequency and the length ratio (the ratio to cancer length) of NE. The secondary endpoints were the relationships between NE and clinicopathological factors, including endoscopic findings of a gastritis-like appearance (GLA), reddish depressed lesion (RDL), and white nodular mucosa (WNM). The frequency and length ratio of the internal surface type in HP-eradicated GCs were significantly higher (82.5% vs 50%, *P* = 0.005) and larger (11.6 ± 10.6 vs 4.2 ± 9.9, P < 0.001) than those in HP-infected GCs, and the increase was more significant according to the passage of time since HP eradication. The frequency and length ratio of marginal surface type and full gland type were not significantly different between the two groups, but the coexistence of internal surface and full gland types was statistically significant (p < 0.001). The frequencies of GLA, RDLs, and WNM in HP-eradicated GCs were significantly higher than those in HP-infected GCs. GLA-positive GCs were covered more widely by internal surface type than GLA-negative GCs (13.3% vs. 6.6%, *P* = 0.003). Various types of NE were noted in gastric cancer, and the internal surface type of NE was shown to be significantly linked to HP-eradicated cancer and GLA.

## Introduction

Previous reports have indicated that *Helicobacter pylori* (HP) eradication reduces gastric cancer (GC) incidence, but GCs are still found after HP eradication [1–3]. The clinicopathological characteristics of HP-eradicated GCs are different from those of HP-infected GCs. The majority of HP-eradicated GCs are small, depressed, and mainly composed of differentiated type adenocarcinoma [4,5], which corresponds to the tubular adenocarcinomas in the classification of Japanese Gastric Cancer Association (JGCA) [6].

HP-eradicated GCs are often difficult to diagnose because their microsurface and microvascular patterns are similar to those of the surrounding inflammatory mucosa on magnifying endoscopy combined with narrow-band imaging (ME-NBI). Kobayashi [7] dubbed the challenging endoscopic finding as a gastritis-like appearance (GLA) and suggested that this finding was due to the surface differentiation of adenocarcinoma. Kitamura [8] reported that epithelium with low-grade atypia (ELA) was frequently present on the surface of HP-eradicated GCs. Saka showed that HP-eradicated GCs frequently accompanied non-neoplastic epithelium (NE), which showed no cytological atypia and thus formed a distinct boundary between cancer tissues [9]. NE, ELA, and surface differentiation are key pathological phenomena that occur in GCs after HP eradication and may cause difficulty in making an endoscopic diagnosis of GC. Another reason for the difficulty may be various mucosal alterations in the background of *H*. *pylori*-eradicated gastric mucosa, which include reddish depressed lesions (RDLs) mimicking small depressed cancers [10], multiple white elevated lesions (MWELs) [11,12], and white nodular mucosa (WNM) that spreads into the adjacent atrophic area in the gastric corpus.

In the present study, we explored the characteristics of NE in detail and clarified the relationships between NE and clinicopathological factors in HP-eradicated and HP-infected GCs.

## Materials and methods

### Study subjects

A total of 432 early GCs resected by endoscopic submucosal resection (ESD) at Nippon Medical School Hospital (Tokyo, Japan) from April 2013 to October 2018 were deemed eligible in the present study. Based on the HP status, the GCs were classified into 122 HP-eradicated GCs, 121 HP-infected GCs, and 189 GCs with indeterminate HP status. We then extracted 80 early GCs (40 HP-eradicated GCs and 40 HP-infected GCs) that were matched for macroscopic type, tumor size, and histological type of adenocarcinoma. Using these 80 matched GCs, we evaluated NE and clinical characteristics.

The HP infection status was assessed as follows: HP-infected GCs were defined as those obtained from patients who had been HP-infected at the time of ESD. HP infection was considered present if at least one of the following tests was positive: ^13^C-Urea Breath Test (UBT, Otsuka, Tokushima, Japan), stool HP antigen test (Premier Platinum HpSA; Meridian Bioscience, Cincinnati, OH, USA), and serum HP IgG antibody (E-plate, Eiken, Tokyo, Japan) with the cut-off level being set at 10. HP-eradicated GCs were defined as those obtained from patients who had a definite history of eradication therapy and had been HP-negative on a UBT or stool antigen test. The passage of time since HP eradication in eligible patients was collected from medical records and analyzed according to the number of months.

The study was conducted in accordance with the Declaration of Helsinki. The study protocol with opt-out consent was approved by the medical ethics committee of Nippon Medical School Hospital (registry no: 30-02-1077). We collected all data from the anonymous clinical data between October 2018 and April 2019. All data were fully anonymized prior to analysis to protect patient privacy.

### Endoscopic evaluations

Eligible patients underwent an endoscopic work-up with a magnifying endoscope (GIF-H260Z, GIF-H290Z; Olympus Medical System, Tokyo, Japan) before ESD. An endoscopic cancer diagnosis with white light imaging and ME-NBI was based on the classification of JGCA [6] and MESDA-G [13]. Tumor location was divided into upper, middle, and lower third of the stomach according to the longitudinal position of the center of the tumor. Macroscopic type was classified according to the JGCA classification, and superficial lesions of 0-IIa, 0-IIb, and 0-IIc of were enrolled in this study. The presence or absence of GLA, RDLs, MWELs, and WNM was retrospectively reviewed by an expert endoscopist blinded to the information on HP infection status of the GCs.

**Fig 1:**
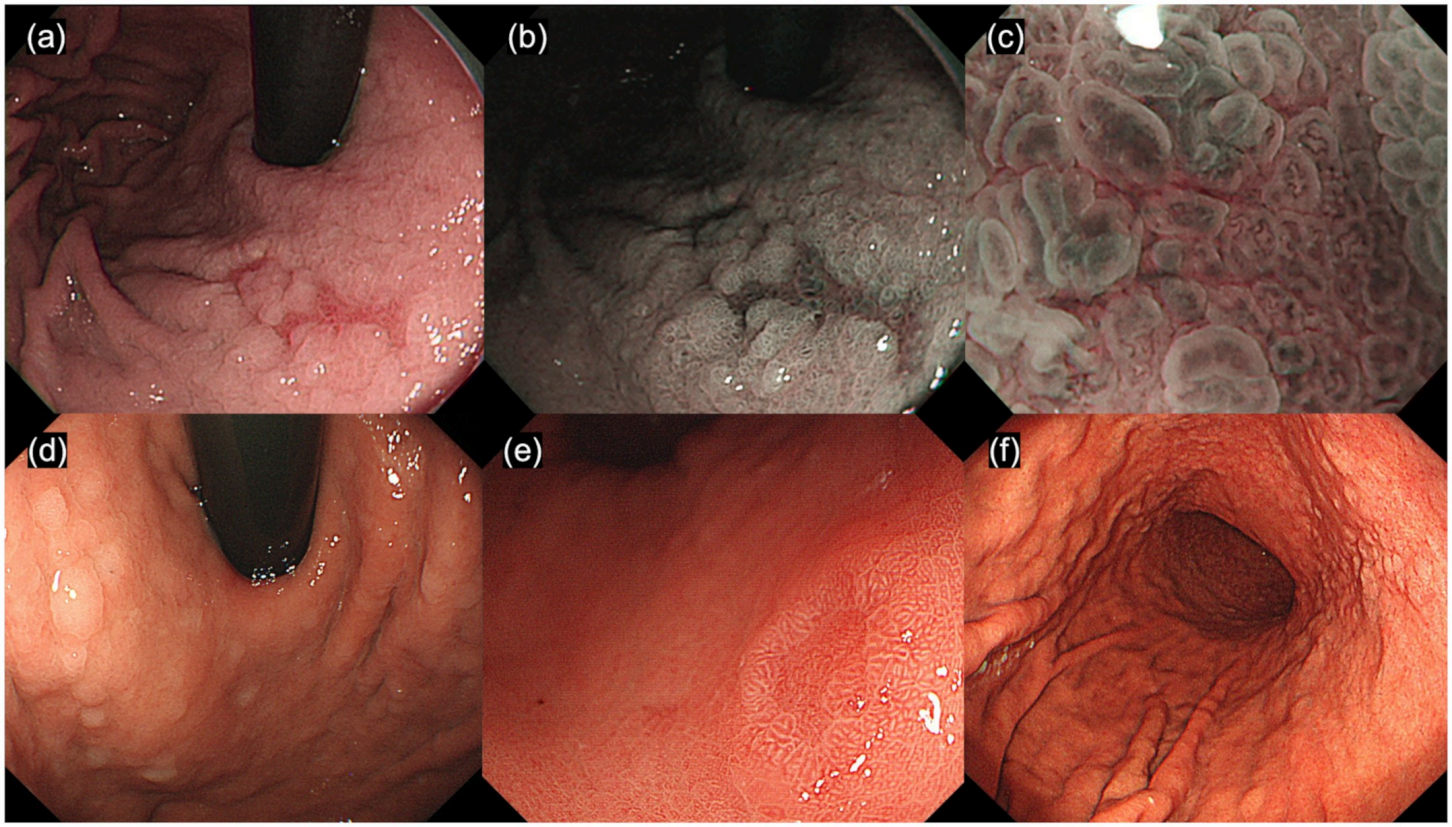
Representative endoscopic images. (a) White-light endoscopic view reveals a gastritis-like appearance (GLA). (b) Narrow-band imaging view with GLA. (c) Narrow-band imaging with magnifying endoscopic view with GLA. (d) White-light endoscopic view reveals multiple white elevated lesions (MWELs). (e) White-light endoscopic view reveals a reddish depressed lesion (RDL). (f) White light endoscopic view reveals a white nodular mucosa (WNM).

GLA was characterized using NBI-ME by uniform papillae and/or tubular pits with a whitish border, regular or faint microvessels and unclear demarcation, resembling the adjacent noncancerous mucosa [7]. RDLs were defined as multiple well-localized shallow depressions, ≤20 mm in diameter, showing clear redness compared to the surrounding mucosa [10]. MWELs were identified as multiple, white and slightly elevated lesions with a smooth surface that was clearly circumscribed [11,12]. We defined WNM as lesions with a white protruding mucosa with an appearance of small multiple nodules, adjacent to the atrophic mucosa in the gastric corpus (Fig 1).

### Histological analyses

All ESD specimens were fixed in formalin, cut into 2-mm-wide strips embedded in paraffin, and stained with hematoxylin-eosin-stained (HE). Histologic type and tumor depth were determined based on the JGCA classification. Cytological atypia grade of GC was classified as high-grade atypia or low-grade atypia based on nuclear features (size and shape). High-grade atypia was defined as nuclei with swelling and irregular shapes. Low-grade atypia was defined by elongated nuclei. Regarding the histological assessment of the background mucosa, we retrospectively evaluated the degree of inflammatory cell infiltration and intestinal metaplasia on HE slides of the ESD specimens. According to the updated Sydney system [14], the degrees of neutrophil and mononuclear infiltration were divided into two categories as follows: normal to mild and moderate to severe. According to the degree of intestinal metaplasia in the surrounding mucosa, gastric cancers were divided into those originating from intestinal metaplasia (GC-IM) and those from gastric mucosa (GC-GM). GC-IM was defined by the presence of intestinal metaplasia on both sides of an ESD specimen surrounding the gastric cancer lesion. GC-GM was defined by the absence of intestinal metaplasia on either side or one side of an ESD specimen surrounding the gastric cancer lesion.

**Fig 2:**
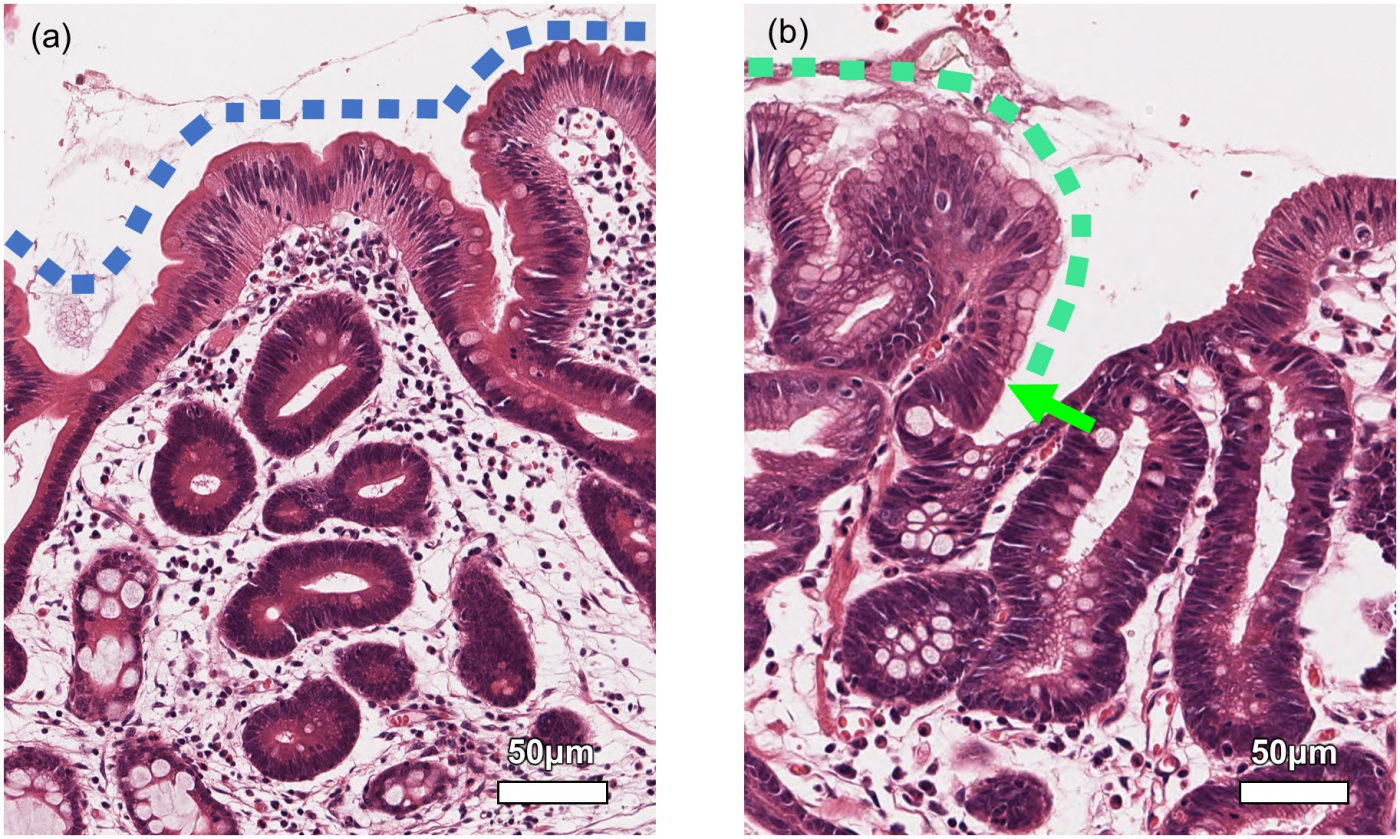
Definition of non-neoplastic epithelium (NE). (a) Columnar epithelium within GC has no atypia (blue dot line). These cells are not defined as NE, because they do not have clear front with surrounding cancer cells. They are thought to be epithelium with low-grade atypia or surface differentiation. (b) Columnar epithelium within GC has no atypia (green dot line). These cells are defined as NE, because they have clear front with surrounding cancer cells (green arrow). (a, b, hematoxylin-eosin, original magnification ×200; scale bar = 50 μm).

**Fig 3:**
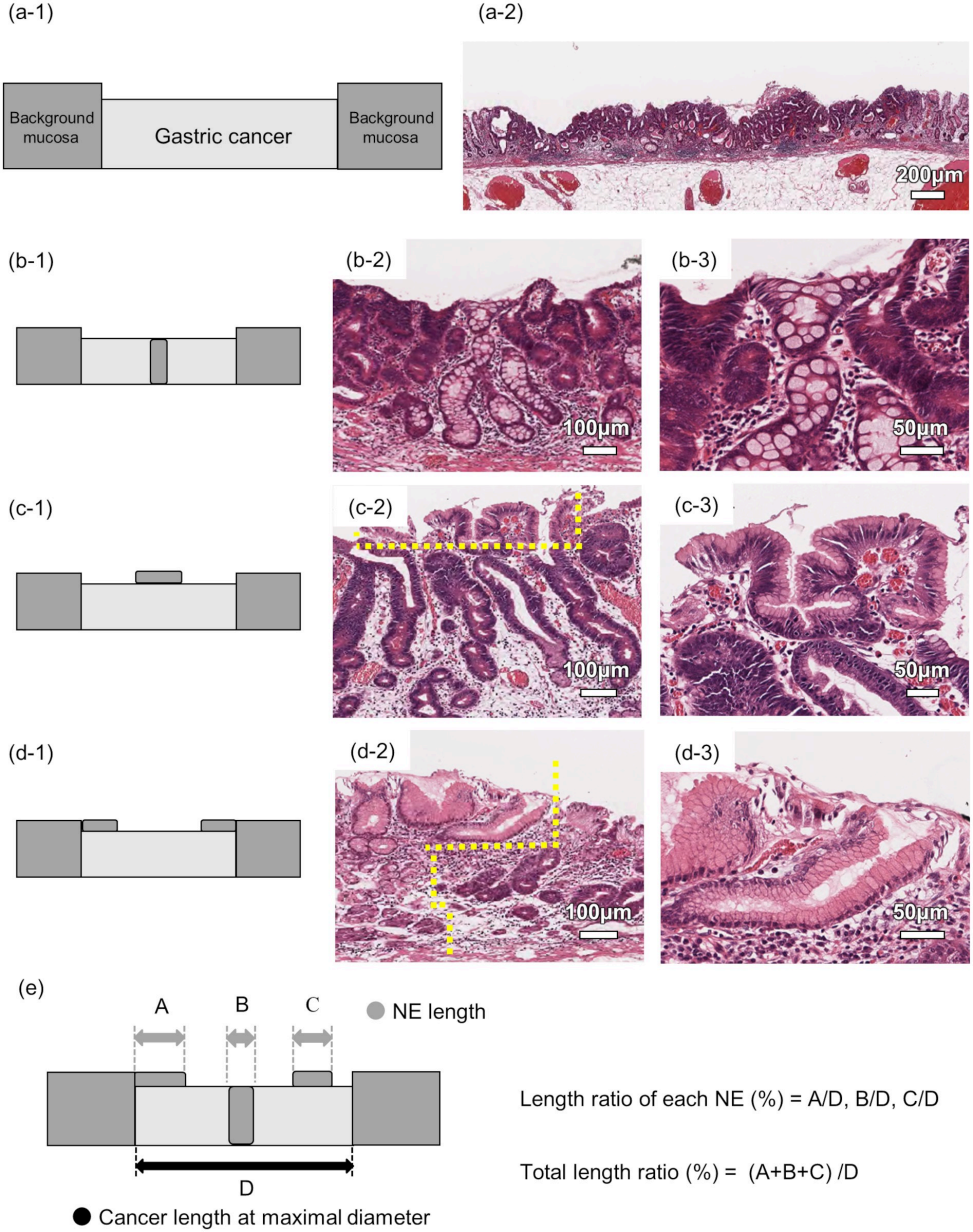
Schematic illustrations and representative micrographs in each type of non-neoplastic epithelium (NE). (a-1) Schematic illustration of gastric cancer (GC) not having NE. (a-2) Representative micrograph of GC not having NE. (b-1) Schematic illustration of full gland type of NE. NE appears from the bottom to the surface of mucosal layer within GC. (b-2) Representative micrograph of full gland type. Micrograph shows a NE gland appears between tubular cancerous glands. (b-3) Micrograph with high-power magnification of full gland type. (c-1) Schematic illustration of internal surface type. NE appears superficially in the internal area of GC. (c-2) Representative micrograph of internal surface type NE. Micrograph shows that NE lies atop tubular cancerous glands. The superficial part of the yellow dotted line represents the field of internal surface type NE. (c-3) Micrograph with high-power magnification of internal surface type. (d-1) Schematic illustration of marginal surface type. NE appears continuously from the background mucosa. (d-2) Representative micrograph of marginal surface type. Micrograph shows that NE continuing from the background mucosa covers the tubular cancerous glands. The left side of the yellow dotted line represents the field of non-cancerous mucosa and the right side represents the field of cancerous mucosa. (d-3) Micrograph with high-power magnification of marginal surface type. (e) The method of measuring the NE length ratio. The NE length ratio is the total length of the NE divided by the length of the cancer measured using the representative histologic section with the maximal cancer diameter, multiplied by 100. (a-2, low power magnification, scale bar = 200 μm; b-2, c-2, d-2, middle power magnification, scale bar = 100 μm; b-3, c-3, d-3, high power magnification, scale bar = 50 μm).

NE was evaluated without any clinical information, including the HP infection status by one investigator. After the evaluation, two reviewers assessed the result together. NE was defined in cases meeting the following criteria: 1) columnar epithelium without cellular atypia on nuclear polarity and shape; 2) columnar epithelium having a clear front between surrounding cancer cells; and 3) columnar epithelium inside or upon cancerous mucosa. This definition was compatible with that reported by Saka [9], but it differed from that used for surface differentiation [7] and ELA [8]. NE was defined as a columnar epithelium with no cellular atypia having a clear front with cancer cells surrounding it (Fig 2). We classified NE into surface type and full gland type. Full gland type referred to non-neoplastic full glands isolated within cancerous tissue with a structure that spread from the bottom to the surface of the mucosa (Fig 3b). Surface type referred to NE located only on the surface of cancerous tissue. Surface type was further divided into internal surface type and marginal surface type; the former was NE inside cancerous mucosa (Fig 3c), and the latter was NE on the margin of cancerous mucosa (Fig 3d). The frequency and length of NE were assessed using the representative histologic section with the largest cancer diameter in each case. The length of NE was calculated as the NE length ratio (%), which is the length of each NE divided by the length of the cancer (Fig 3e) measured using the representative histologic section with the largest cancer diameter.

### Statistical analyses

All statistical analyses were performed using the EZR software program (Saitama Medical Center, Jichi Medical University) [15]. We analyzed the baseline characteristics and outcomes of this study using the Mann-Whitney U test for continuous variables, the chi-square test or Fisher’s exact test for categorial variables, and Tukey’s post hoc test was used for multiple comparisons. A *P*-value of <0.05 was considered to indicate a statistically significant difference.

## Results

### Comparison of clinicopathological features between HP-eradicated GC and HP-infected GC

There were no significant differences in the tumor size, macroscopic type, histologic type, or cytological atypia between HP-infected and HP-eradicated GCs (Table 1). Inflammatory cell infiltration, by both neutrophils and mononuclear cells, was significantly marked in HP-infected GCs in comparison to HP-eradicated GCs. When evaluating the existence of NE, the internal surface type was found to be significantly more frequent in HP-eradicated GCs than in HP-infected GCs (82.5% vs. 50.0%, *P* = 0.005), whereas the frequency of the other two types (marginal surface type and full gland type) did not differ markedly between the two GC groups. Regarding endoscopic findings, the frequencies of GLA, RDL, and WNM in HP-eradicated GCs were significantly higher than those in HP-infected GCs. There were no significant differences in MWELs between the two GC groups.

**Table 1:** Clinicopathological features in HP-infected and HP-eradicated GCs.

	HP-infected GCs (n = 40)	HP-eradicated GCs (n = 40)	*P* value
Tumor characteristics			
Tumor location (Upper third/Middle third/Lower third)	20/13/7	16/18/6	0.52
Tumor size (mm), mean ± SD	12.7 ± 7.0	12.6 ± 6.9	1.0
Macroscopic type (0-IIa/0-IIb/0-IIc)	14/1/25	14/1/25	1.0
Histologic type (tub1/tub2)	39/1	39/1	1.0
Tumor depth (T1a/T1b)	35/5	39/1	0.20
Cytological atypia (high/low)	20/20	21/19	1.0
Background mucosa characteristics			
Neutrophilic inflammation (normal to mild/moderate to severe)	25/15	40/0	<0.001
Mononuclear cell inflammation (normal to mild/moderate to severe)	14/26	30/10	<0.001
Existence of endoscopic findings, no. (%)			
GLA	3 (7.5)	13 (32.5)	0.01
MWEL	7 (17.5)	5 (12.5)	0.75
RDL	2 (5.0)	16 (40.0)	<0.001
WNM	20 (50.0)	30 (75.0)	0.04
Existence of NE, no. (%)			
Marginal surface type	17 (43.5)	23 (57.5)	0.26
Internal surface type	20 (50.0)	33 (82.5)	0.005
Full gland type	11 (27.5)	19 (47.5)	0.11
Two or three of three types	14 (35.0)	28 (70.0)	0.002
Length ratio of NE, mean ± SD			
Marginal surface type	3.1 ± 4.5	7.0 ± 11.2	0.13
Internal surface type	4.2 ± 9.9	11.6 ± 10.6	<0.001
Full gland type	2.6 ± 5.9	2.6 ± 4.3	0.19
Total length of three types	9.9 ± 11.3	21.2 ± 16.7	<0.001

HP, *Helicobacter pylori*; GC, gastric cancer; SD, standard deviation; GLA, gastritis-like appearance; MWEL, multiple white elevated lesions; RDL, reddish depressed lesion; WNM, white nodular mucosa; NE, non-neoplastic epithelium.

**Fig 4:**
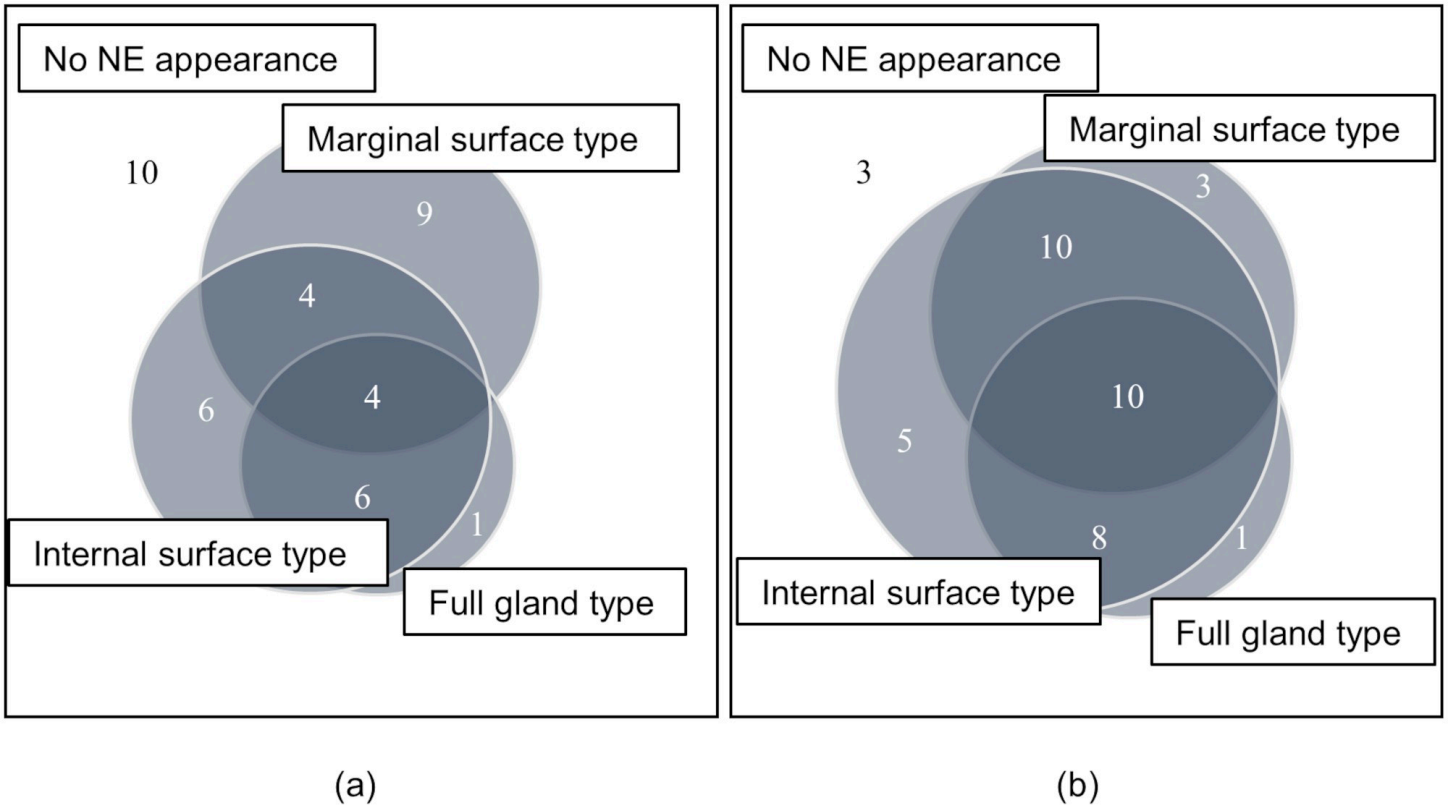
Venn diagrams depicting occurrence patterns of NE in the infection group and the eradication group. (a) *Helicobacter pylori* (HP)-infected GCs. (b) HP-eradicated GCs.

As shown in the Venn diagram of Fig 4 and Table 1, the frequency of the coexistence of 2 or 3 NE types in HP-eradicated GCs (28 of 40, 70%) was significantly (*P* = 0.002) higher than the frequency in HP-infected GCs (14 of 40, 35%). The results of the length ratio of NE were similar to those of the frequency of NE. The length ratio of internal surface type in HP-eradicated GCs was significantly larger than that in HP-infected GCs, but the length ratios of full gland type and marginal surface were not markedly different between the two GC groups (Table 1).

### Factors related to internal surface type NE

Of the three types of NE, only the internal surface type was significantly related to HP-eradicated GCs. We next evaluated the factors linked to the presence of internal surface type NE (Table 2). The presence and length of full gland type NE were significantly (*P* < 0.001) related to the presence of internal surface type. The tumor depth, histologic type, cytological atypia, marginal surface type, and endoscopic findings (data not shown) were not related to the internal surface type.

**Table 2:** Comparison of the clinicopathological characteristics between internal surface type NE-positive and internal surface type NE-negative GCs.

	internal surface NE-positive GCs (n = 53)	internal surface NE-negative GCs (n = 27)	*P* value
HP status (infection/eradication)	20/33	20/7	0.005
Tumor depth (M/SM)	51/2	23/4	0.17
Histologic type (tub1/tub2)	52/1	26/1	1.0
Cytological atypia (high /low)	24/29	17/10	0.16
Existence of NE, no. (%)			
Marginal surface type	28 (52.8)	12 (44.4)	0.64
Full gland type	28 (52.8)	2 (7.4)	<0.001
Length ratio of NE, mean ± SD			
Marginal surface type	4.7 ± 7.4	5.7 ± 11.0	0.96
Full gland type	3.1 ± 4.5	1.5 ± 6.2	< 0.001

NE, non-neoplastic epithelium; GC, gastric cancer; HP, *Helicobacter pylori*; SD, standard deviation.

### Relationship between NE and GLA

The comparison of NE between GLA positive (16 cases) and negative (64 cases) groups showed that the presence of GLA was associated with the frequency of internal surface type (14 of 16, 87.5%; vs, 39 of 64, 60.9%; *P* = 0.07), and GLA-positive GCs were covered more widely by internal surface type NE than GLA-negative GCs (13.3% vs. 6.6%, *P* = 0.003). However, the frequencies and length ratios of marginal surface type and full gland type did not differ between GLA-positive and GLA-negative GCs.

### Relationship between NE and the degree of intestinal metaplasia in the mucosa surrounding GC

Next, we investigated the relationship between NE and the mucosa surrounding GC. According to the histological features of the mucosa around the GC lesion, GCs were classified into GC-IM originating from intestinal metaplasia and GC-GM originating from gastric mucosa. We next compared clinicopathological features between GC-IM and GC-GM (Table 3). The HP infection status (current infection/after eradication) did not differ between GC-IM and GC-GM. The tumor depth of GC-GM was significantly deeper in comparison to GC-IM. The frequency of full gland type NE in GC-IM (51.5%) was significantly (p = 0.037) higher in comparison to GC-GM (27.7%), whereas the frequencies of internal surface type and marginal surface type did not differ between GC-IM and GC-GM.

**Table 3:** Comparison of the clinicopathological characteristics between GC-IM and GC-GM.

	GC-IM (n = 33)	GC-GM (n = 47)	*P* value
*Helicobacter pylori* status (current infection/after eradication)	19/14	21/26	0.36
Tumor characteristics			
Tumor location (Upper third/Middle third/Lower third)	3/17/13	10/14/23	0.11
Tumor size (mm), mean ± SD	13.3 ± 7.2	12.1 ± 6.7	0.47
Macroscopic type (0-IIa/0-IIb/0-IIc)	8/0/25	20/2/25	0.08
Tumor depth (T1a/T1b)	33/0	41/6	0.039
Existence of NE, no. (%)			
Marginal surface type	17 (51.5)	23 (48.9)	1.0
Internal surface type	25 (75.8)	28 (59.6)	0.16
Full gland type	17 (51.5)	13 (27.7)	0.037
Length ratio of NE, mean ± SD			
Marginal surface type	3.4 ± 5.2	6.2 ± 10.4	0.55
Internal surface type	10.5 ± 14.0	6.1 ± 7.6	0.19
Full gland type	3.2 ± 5.0	2.2 ± 5.4	0.059

GC-IM, gastric cancer originating from intestinal metaplasia; GC-GM, gastric cancer originating from gastric mucosa; NE, non-neoplastic epithelium; SD, standard deviation.

In order to investigate why full gland type NE was frequent in GC-IM, the relationship between the two types of GCs and full gland type NE was further examined in detail. We subclassified full gland type NE into intestinal type (composed of intestinal metaplasia) and gastric type (composed of a gastric gland without intestinal metaplasia). As shown in Table 4, the intestinal type of full gland NE was frequently observed in GC-IM originating from intestinal metaplasia, whereas the gastric type of full gland NE was frequently observed in GC-GM originating from gastric mucosa without intestinal mucosa. This result indicated that the histological nature of full gland type NE in GC corresponded to the nature of the mucosa surrounding GC. In this study cohort, GC-GM (47/80 = 59%) was dominant in comparison to GC-IM (33/80 = 41%), and thus it was hypothesized that the gastric type of full gland NE would be dominant in comparison to the intestinal type. However, the intestinal type (19/80, 23.8%) was dominant in comparison to the gastric type (11/80, 13.8%), suggesting that the columnal gland composed of intestinal metaplasia could more easily remain in gastric cancer tissue as the full gland type of NE.

**Table 4:** Comparison of full gland type NE between GC-IM and GC-GM.

	Full gland type NE		
	Presence (n = 30, 37.5%)		Absence (n = 50, 62.5%)
	Intestinal type (n = 19, 23.8%)	Gastric type (n = 11, 13.8%)	
GC-IM (n = 33)	14	3	16
GC-GM (n = 47)	5	8	34

NE, non-neoplastic epithelium; GC-IM gastric cancer originating from intestinal metaplasia; GC-GM, gastric cancer originating from the gastric mucosa.

### Relationship between NE and passage of time since HP eradication

The passage of time since eradication therapy was known in detail in 30 out of 40 HP-eradicated GC patients; the average time was 26 months (range 6–180 months). We divided the 30 patients into two categories: the early onset group (passage of time since eradication < 60 months [5 years]) and the late onset group (time > 60 months). The comparison of clinicopathological features between the early and late onset groups is shown in Table 5. The frequency of internal surface type NE in the late onset group tended to be higher than in the early onset group (100% vs. 68.4%, P = 0.061). The late onset group was significantly associated with a higher rate of internal surface type (14.8% vs. 7.9%, P = 0.042), but not with marginal surface type or full gland type. There were no significant differences between the two groups with regard to tumor location, tumor size, macroscopic type, and tumor depth.

**Table 5:** Comparison of the clinicopathological characteristics between early and late onset after HP eradication groups in 30 HP-eradicated GC.

	Early onset group (n = 19)	Late onset group (n = 11)	*P* value
Tumor characteristics			
Tumor location (Upper third/Middle third/Lower third)	3/9/7	3/4/4	0.79
Tumor size (mm), mean ± SD	14.6 ± 6.7	12.4 ± 6.0	0.31
Macroscopic type (0-IIa/0-IIb/0-IIc)	9/2/8	4/0/7	0.43
Tumor depth (T1a/T1b)	19/0	10/1	0.37
Existence of NE, no. (%)			
Marginal surface type	9 (47.3)	7 (63.6)	0.47
Internal surface type	13 (68.4)	11 (100)	0.061
Full gland type	8 (42.1)	6 (54.5)	0.71
Length ratio of NE, mean ± SD			
Marginal surface type	3.7 ± 5.5	6.5 ± 9.0	0.39
Internal surface type	7.9 ± 9.4	14.8 ± 12.0	0.042
Full gland type	1.9 ± 3.3	2.5 ± 2.9	0.47

NE, non-neoplastic epithelium; GC, gastric cancer; HP, *Helicobacter pylori*; SD, standard deviation.

## Discussion

NE has been observed within GC tissues in various patterns. Therefore, we classified NE into three types: full gland type, internal surface type, and marginal surface type, according to the location and structure. Using this classification, we showed that the internal surface type was observed more frequently and widely in HP-eradicated GCs than in HP-infected GCs. The increase of the internal surface type became more significant according to the passage of time since HP eradication. Although the marginal surface type and full gland type did not show a statistically significant linkage to HP-eradicated GCs, the combined presence of two or three NE types was more frequent in HP-eradicated GCs than in HP-infected GCs.

The origin of internal surface type NE and the mechanism underlying its frequent and long presence in HP-eradicated GCs are unknown. One possible origin of internal surface type NE is cancer cells themselves. However, while the origin of ELA can be attributed to the surface differentiation of cancer cells themselves, NE has a distinct border with cancerous tissue and thus may not be derived from cancer cells by surface differentiation. Another possible origin is non-neoplastic glands that exist adjacent to the internal surface type NE. Indeed, the present study showed a very strong linkage between the existence of full gland type NE and internal surface type NE (p < 0.001), indicating the notable coexistence of the two types of NE. During cancer progression, some non-neoplastic glands may remain within gastric cancerous tissue, regardless of the HP status, as shown in this study. HP eradication may thus somehow stimulate the foveolar proliferation of the isolated non-neoplastic glands, which then spread over cancerous tissue and become recognized as internal surface type NE. Previous studies have shown that MUC5AC, which is specific for foveolar epithelium, was decreased by HP infection and increased by HP eradication [16–18]. Hyperplastic changes in the foveolar epithelium, such as multiple whitish flat elevated lesions (MWFL) [19], are frequently observed after HP-eradication [11]. In the present study, the frequency of WNM related to foveolar hyperplasia was significantly higher in HP-eradicated GCs than in HP-infected GCs. Taken together, these findings suggest that HP eradication induces the proliferation of foveolar epithelium of non-neoplastic gastric glands in GCs, which may lead to the frequent appearance of internal surface type NE in HP-eradicated GCs over time.

Some HP-eradicated GCs show microsurface and microvascular patterns similar to the surrounding inflammatory mucosa, hampering an endoscopic cancer diagnosis. Kobayashi et al. [7] initially reported this challenging endoscopic finding as GLA and found that GLA was more frequent in HP-eradicated GCs than in HP-infected GCs and significantly correlated with histological surface differentiation. Saka reported a significant relationship between GLA and NE. NE covering over 10% of the cancerous area was observed in more than 90% of GCs showing GLA but in only 12.5% of GCs without GLA. The results of the present study were comparable to those of these previous studies, and we showed that internal surface NE was significantly related to GLA, while the other two type of NE were not. The total length of NE was significantly longer in GLA-positive GCs than in GLA-negative GCs, but the length ratio of NE in GLA-positive GCs was 24.3% in our study. Therefore, NE may be a partial cause of GLA, and the combination of NE and surface differentiation or ELA may be attributable to GLA.

In this study, we further examined full gland type NE using the classification of intestinal type and gastric type. As a result, the intestinal type of full gland NE was frequently observed in GC-IM originating from intestinal metaplasia, whereas the gastric type of full gland NE was frequently observed in GC-GM originating from gastric mucosa without intestinal metaplasia. This result suggested that the full gland type NE might be a non-neoplastic gland that remains within gastric cancerous tissue during cancer progression. Interestingly, the intestinal metaplasia type of full gland NE was dominant in comparison to the gastric type whereas GC-IM was not dominant in comparison to GC-GM in this study cohort, indicating that intestinal metaplasia could more easily remain in gastric cancer tissue as the full gland type of NE.

Several limitations associated with the present study warrant mention. First, this study was a single-center retrospective study using 40 HP-infected and 40 HP-eradicated GCs extracted from 243 GCs resected by ESD. We speculated that macroscopic type, cancer size, and histologic type of cancer might affect the features of NE, so we extracted these 80 GCs by 1:1 matching for these factors. As a result, most of the study subjects demonstrated small GCs mainly composed of well-differentiated adenocarcinoma (tub1). As such, whether or not poorly differentiated adenocarcinoma or mixed type of adenocarcinoma show a similar behavior with regard to NE is unclear. Further large-scale research will be necessary in the future, because the number of enrolled patients in the present study was relatively small. Second, we evaluated NE but not the surface differentiation or ELA, which is reported to be involved in GLA. The most obvious difference between NE and surface differentiation or ELA is the presence or absence of a front caused by the difference in cellular atypia between cancer cells and non-neoplastic cells. This front is relatively easy to identify, and the recognizability of NE based on this front may not differ significantly among investigators. Conversely, in some GCs, it may be difficult to identify the surface differentiation or ELA. In GCs with high-grade atypia, surface differentiation or ELA may be recognizable by the gradual alteration from the part with high-grade atypia to the surface part with low-grade atypia. However, in GCs with low-grade atypia, surface differentiation or ELA may be difficult to identify, as almost all cancerous tissues show low-grade atypia, and its rate of recognition may differ among investigators. GCs with low-grade atypia accounted for about half of the GCs enrolled in this study, for these reasons, we evaluated only NE and not surface differentiation or ELA. In conclusion, various types of NE were detected in GCs, and internal surface type NE, which may be derived from full gland type NE, was found to be an essential histological alteration of HP-eradicated GCs. The presence of internal surface type NE may be partially responsible for the difficulty in making an endoscopic cancer diagnosis.
